# Customizing the reduction of individual graphene oxide flakes for precise work function tuning with meV precision†

**DOI:** 10.1039/d0na00321b

**Published:** 2020-06-01

**Authors:** Yuefeng Huang, Dengke Ma, Patrick Turner, Gavin E. Donnelly, Joel M. Katzen, William R. Hendren, J. Marty Gregg, Robert M. Bowman, Lifa Zhang, Gang Zhang, Fumin Huang

**Affiliations:** School of Mathematics and Physics, Queens University Belfast Belfast BT7 1NN UK f.huang@qub.ac.uk; NNU-SULI Thermal Energy Research Center (NSTER), Center for Quantum Transport and Thermal Energy Science (CQTES), School of Physics and Technology, Nanjing Normal University Nanjing 210023 China; Institute of High Performance Computing, A*STAR Singapore 138632 Singapore

## Abstract

Being able to precisely control the reduction of two-dimensional graphene oxide films will open exciting opportunities for tailor-making the functionality of nanodevices with on-demand properties. Here we report the meticulously controlled reduction of individual graphene oxide flakes ranging from single to seven layers through controlled laser irradiation. It is found that the reduction can be customized in such a precise way that the film thickness can be accurately thinned with sub-nanometer resolution, facilitated by extraordinary temperature gradients >10^2^ K nm^−1^ across the interlayers of graphene oxide films. Such precisely controlled reduction provides important pathways towards precision nanotechnology with custom-designed electrical, thermal, optical and chemical properties. We demonstrate that this can be exploited to fine tune the work function of graphene oxide films with unprecedented precision of only a few milli electronvolts.

## Abstract 2

Laser-controlled reduction of individual graphene oxide films provide unprecedented work function tuning with millivolt precision.

## Graphical abstract

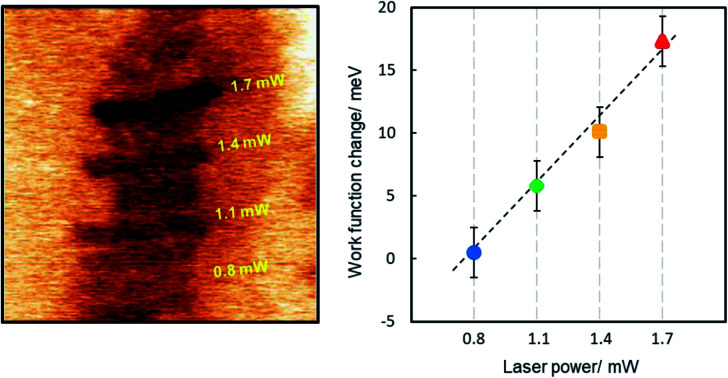

Graphene oxide (GO) films are oxidized graphene sheets decorated with various oxygen functional groups, such as epoxide and hydroxyl groups on the carbon basal plane and carbonyl and carboxyl groups attached to the edges.^1–5^ Due to the non-stoichiometric nature of the structure, the thickness of a monolayer GO sheet is not well defined, varying roughly between 0.8 and 1.3 nm, dependent on the density and species of functional groups.^1–5^ The functional groups strongly impact the GO properties, making them drastically different from those of graphene. As is well known, graphene is one of the most conductive materials electrically and thermally.^6,7^ By contrast, GO is an insulator both for electricity and heat.^1–5^ The transport of electrons and phonons within GO is severely hindered by high-density disordered sp^3^ domains and the defects of carbon vacancies. Graphene is hydrophobic, insoluble in water, while GO is amphiphile with a largely hydrophobic basal plane and hydrophilic edges, hence can be well dispersed in water and many other solutions.^3–5^ One important application of GO is being used as the precursor of graphene. GO can be reduced to graphene-like films by removing functional groups through a variety of techniques, including chemical, thermal, optical and electrical methods.^2,8–12^ After reduction, the electrical conductivity can be improved by several orders of magnitude, comparable to that of conductive polymers, making them an important building block in flexible electronics.^11,13^ As GO is dispersible in water, aqueous GO solutions can be conveniently processed on a large-scale for industrial applications, *e.g.*, through inkjet printing and spray coating, which then can be reduced to graphene-like films, enabling a broad range of applications that otherwise are difficult to achieve with graphene (as it is hydrophobic), such as anti-electrostatic coating, corrosion–protection layers, and transparent conductors.^1–5,14–17^ Apart from being the precursor of graphene, research on individual GO nanofilms is immensely interesting in its own right. Through programmed laser irradiation, complex patterns can be structured on individual GO films to develop transparent and flexible electronics.^18–20^ Apart from the electrical properties, the optical, thermal and chemical properties of GO films all have been demonstrated to be easily manipulated by reduction;^2,8–12^ therefore, precisely controlled reduction of individual GO films could have tremendous impacts on a wide variety of technologies.

Here we report the precision reduction of individual GO flakes, ranging from monolayer up to seven layers. Through controlled laser irradiation, we demonstrate that reduction can be controlled in such a meticulous way that the film thickness can be thinned with sub-nanometer resolution. The reduction is caused by extraordinary temperature gradients across the interlayers of GO films, which can be >10^2^ K nm^−1^ under the irradiation of only a few milliwatts laser power. Such precision reduction opens pathways to tailor-make on-demand electrical, chemical and optical properties. We demonstrate that the technique can be exploited to fine tune the work function (WF) of GO films with unprecedented precision in the order of meV. As WF is a fundamental metric of optoelectronic devices, central to the performance of photovoltaics, photodetectors, and light emitting diodes *etc*, being able to accurately tune the WF will have significant implications in device performance.

## Results

**Fig. 1:**
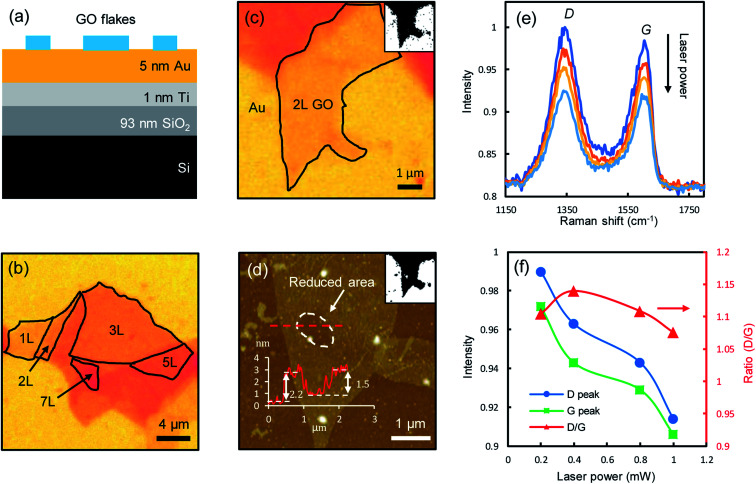
Schematic and optical micrographs of samples. (a) Schematic of the sample structure. (b) Optical micrograph of few-layer GO films on a 5 nm Au substrate. (c) Optical micrograph of a bilayer GO film. Inset: converted black/white image with enhanced contrast. (d) AFM topographic image of the bilayer GO film. Inset (left-bottom): step height profile along the dashed red line through the laser-irradiated spot. Inset (top-right): converted black/white AFM image with enhanced contrast. The reduced area is shown as the white spot. (e) Measured Raman spectra after the spot was successively irradiated by a range of laser powers (from top to bottom), 0.2, 0.4, 0.8, and 1.0 mW, respectively. Each irradiation lasts for 60 seconds. (f) Intensities of the D-mode (circles) and G-mode (squares) and their ratios (triangles).

Fig. 1a shows the schematic of the sample structure. An aqueous GO solution was dispersed (see Methods for details) on a multilayer planar structure, composed of 5 nm Au, 1 nm Titanium adhesion layer, 93 nm SiO_2_ and a Si substrate. The stratified structure is employed to enhance the GO visibility as well as being used as a heat sink. GO films are highly transparent (the absorbance of monolayer GO in the visible range is below 2%,^8^ about one order of magnitude smaller than that of graphene^21^) and difficult to visualize on conventional substrates, *e.g.*, under the 100× objective, on bare SiO_2_/Si substrates the contrast is only about 3% for monolayers, while on the stratified substrate, the contrast is doubled to ∼6%, making the few-layer GO films much easier to visualize (Fig. 1b). The layer number of GO films can be accurately determined with optical contrast spectroscopy (ESI Fig. S1†),^22^ which is shown to be a good indicator of the fraction of sp^2^ hybridized carbon.^23^ GO has very small thermal conductivities around 3 Wm^−1^ K^−1^ at room temperature,^24^ which can reach temperatures of thousands of degrees under strong laser irradiation.^25^ When directly deposited on thermally insulating substrates such as SiO_2_, the irradiated spots could be over heated to fracture the film.^8^ The underlying thin Au film acts as a heat sink to dissipate the heat, allowing the reduction to take place with control.

One of the most notable features of reduced GO films is the decrease of film thickness, as a direct consequence of the detachment of functional groups.^18,20^ To illustrate this effect, we successively irradiated one spot on a bilayer GO film (Fig. 1c) with incrementally increased laser powers (532 nm, 0.2, 0.4, 0.8 and 1 mW, corresponding to a fluence of 7.0 × 10^4^, 1.4 × 10^5^, 2.8 × 10^5^, 3.5 × 10^5^ W cm^−2^, respectively, with the diameter of laser spots of about 600 nm focused by using a 100× objective, for a duration of 60 s of each laser power.) and measured the Raman spectra at the spot right after each irradiation. The atomic force microscopy (AFM) topographic image clearly shows a dent at the irradiated spot (circled area, Fig. 1d), indicating a decrease in film thickness. The original film thickness is ∼2.2 nm. After multiple irradiations, the remaining film thickness is ∼0.7 nm, which is roughly the thickness of two stacking graphene films (0.34 nm for monolayer graphene), indicating that the functional groups attached to the basal graphene plane at the irradiated spot are almost completely removed. The Raman spectra measured at the irradiated spots show the typical D-modes (1345 ± 5 cm^−1^) and G-modes (1604 ± 5 cm^−1^) of GO films (Fig. 1e), in good agreement with the literature.^4,15,26^ The peak positions remain mostly unchanged during the reduction, but the intensities decrease notably with increasing laser powers, with a slight drop in the intensity ratio between the D-mode and G-mode (Fig. 1f). As the D-mode is an indication of the degree of disorder in the GO film, the reduced D/G intensity ratio suggests that the reduced GO films have improved structural orderliness due to the removal of randomly distributed functional groups, which is also confirmed by the decreased full-width-at-half-maximum (FWHM) of the D-modes and G-modes (ESI Fig. S2†). The characteristic 2D peak (around 2700 cm^−1^) of pristine graphene is not observed, as a significant amount of nanoscale defects still remain on the reduced GO films.^9,27^

**Fig. 2:**
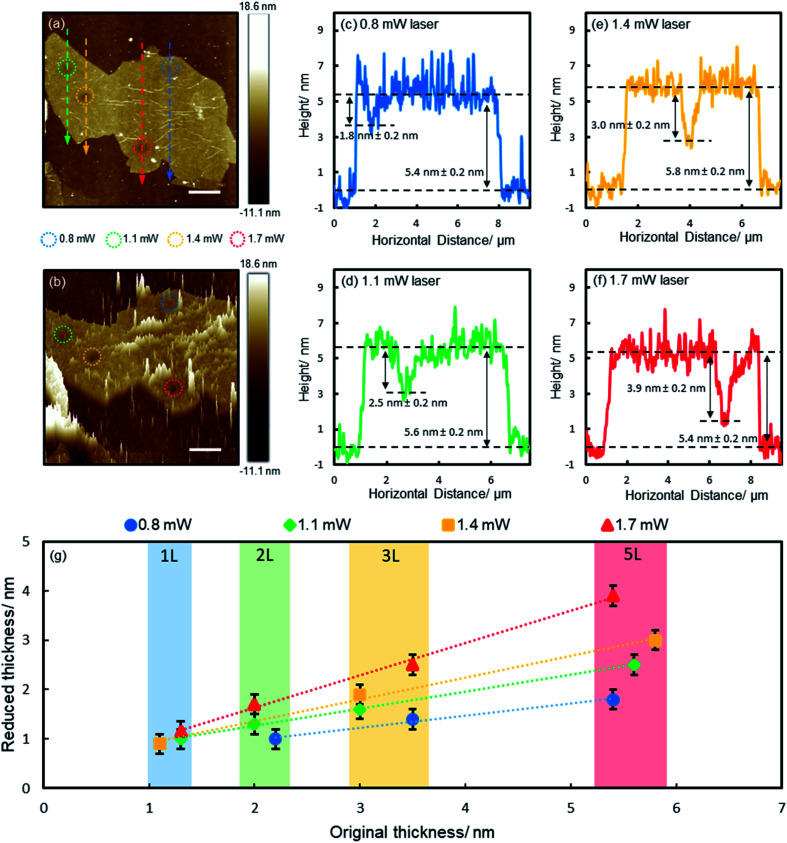
Reducing GO films with laser irradiation. (a) AFM topographic image of a 5-layer GO film. The irradiated spots are marked by circles. Scale bar: 2 μm. (b) 3D plot of (a). (c–f) AFM height profiles along the dashed lines in (a). (g) Reduced film thickness *vs.* the original GO film thickness. Shaded areas indicate the thickness range of each flake.

To gain a deep understanding of the reduction process, we irradiated a range of films of various thicknesses (1, 2, 3 and 5 layers) with different laser powers (0.8, 1.1, 1.4 and 1.7 mW, with corresponding fluences of 2.8 × 10^5^, 3.9 × 10^5^, 4.9 × 10^5^ and 6.0 × 10^5^ W cm^−2^ respectively, each for 60 s) and characterized the reduction effects with AFM. Fig. 2a shows the results of a 5-layer film. The irradiated spots (encircled) appear darker as a result of reduced thickness, which are more obvious on the 3D plot (Fig. 2b). The spikes on the 3D plot correspond to wrinkles and surface contaminants. The reduced spots are clean and smooth, free of spikes, suggesting surface contaminants were detached with functional groups. Fig. 2c–f show the height profiles along the lines across the irradiated spots (Fig. 2a). Apparent dips are seen at the irradiated spots, which are deeper for higher laser powers due to more pronounced reduction effects (Fig. 2c–f). The results of the reduced film thickness in relation to the original film thickness are summarized in Fig. 2g (the AFM images and height profiles of 1–3 layers are shown in ESI Fig. S3–S5†). For all laser powers, the reduced thickness is linearly proportional to the original film thickness (there is some variance in the thickness of each GO flake due to the inhomogeneity of functional groups, which is indicated by the shaded areas in Fig. 2g). However, the gradient is different for different laser powers, including 0.25, 0.34, 0.44 and 0.65 for 0.8, 1.1, 1.4 and 1.7 mW, respectively. Obviously, higher powers produce more pronounced reduction. One exception is for the monolayer film, which shows no notable change when irradiated by using a 0.8 mW laser (ESI Fig. S3†). Here we show that the controlled laser irradiation can manipulate the reduction of individual GO films in such a precise way that the film thickness can be accurately thinned with sub-nanometer precision.

**Fig. 3:**
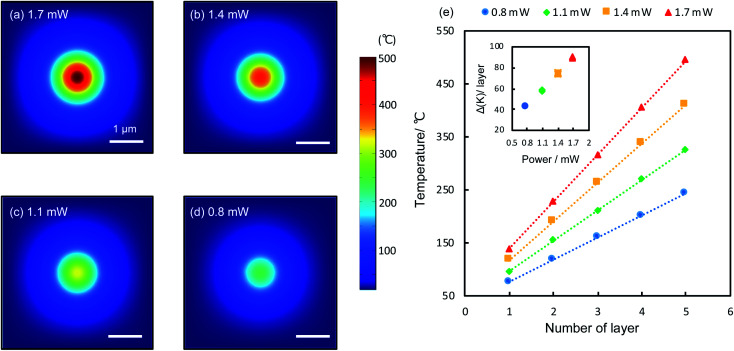
Calculated temperature distribution in a 5-layer GO film. (a–d) Calculated temperature distribution at the top surface of a 5-layer GO film, irradiated by various laser powers as indicated. (e) Temperatures at the different layers (5 refers to the top layer, 1 refers to the bottom layer) within a 5-layer GO film, irradiated by different laser powers. Inset: temperature gradients.

The reduction is caused by raised temperatures at the laser spots. GO has low thermal conductivities both along the in-plane and out-of-plane directions.^28^ Upon the irradiation of the laser, heat is generated and confined within the laser spots, raising the local temperature significantly. According to theoretical calculations (details see Methods), the temperature at the top surface of a 5-layer film can reach 243 °C to 494 °C for laser powers between 0.8 and 1.7 mW. Heat is strongly confined within the laser spots (Fig. 3a–d), consistent with experiments (Fig. 2a and b). Across the GO layers, the temperature drops linearly from the top to the bottom. Fig. 3e shows the calculated temperature (5 refers to the top layer and 1 refers to the bottom layer attached to the Au substrate) within a 5-layer GO film irradiated by various laser powers. For each laser power, the temperature distribution in the GO film is linear, as expected from a homogenous medium. Higher laser powers produce higher temperatures deep into the GO film with larger temperature gradients (inset, Fig. 3e), therefore it can reduce the film more substantially. This is in line with the observed trends of the reduced film thickness shown in Fig. 2g. Temperature plays a central role in the photothermal reduction of GO films. Generally different functional groups have different bonding energies and thus will be dissociated at different temperatures.^2,8–12^ However, apart from the strongly bonded carbonyl groups at the edges, most functional groups attached to the basal plane can be removed at temperatures above 200 °C,^2,29^ which is achievable under most experimental conditions, as indicated by the calculations (Fig. 3e), so that the top few layers would be thoroughly reduced (the only exception is the case of the monolayer film irradiated by using a 0.8 mW power laser, which did not show notable effect of reduction. It could be the temperature being too low, as laser power is very modest and the film is directly in touch with the underlying Au film, with most of the heat being dissipated away). A remarkable temperature gradient of 90 K per layer is achievable with the irradiation of 1.7 mW (inset, Fig. 3e), which can be readily improved to several hundred degrees per nanometer with increased laser powers. Such a system of extraordinary temperature gradients customizable with laser powers, can be exploited as important platforms for a wide range of nanoscale thermodynamics investigations, such as the heat transport across a nanoscale distance and the thermoelectrics of nanomaterials.^30–33^

**Fig. 4:**
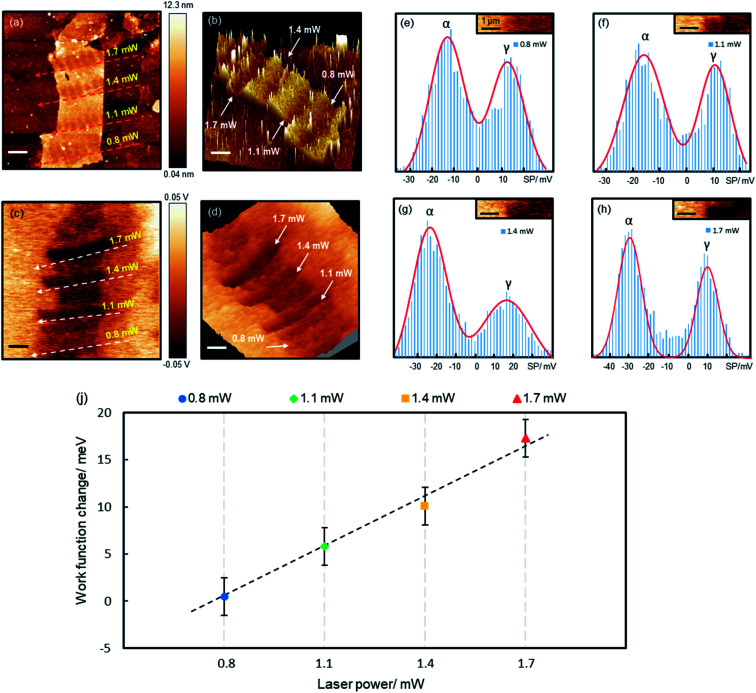
Fine tuning the WF of a 7-layer GO film through laser-controlled precision reduction. (a and b) Topographic and (c and d) SP images of the GO film irradiated by different powers of laser. Scale bars, 2 μm. (b) and (d) Are the corresponding 3D plots of (a) and (c). (e–h) Histogram analysis of the measured SP images, which are fitted with two Gaussian peaks for the reduced GO (α) and Au films (γ). Insets: selected sample areas for the histogram analysis. (i) Changes of WF induced by laser reduction.

Precision reduction opens opportunities for custom-designing the properties of GO films, such as tuning the electrical conductivity, optical band gap and WF. Here we demonstrate that the precisely controlled reduction can be used to fine tune GO's WF. Fig. 4 shows a piece of a 7-layer GO film (6.9–8.6 nm) reduced by various laser powers. For each laser power, a number of spots were irradiated, each for 60 s, separated by about 0.8 μm. We simultaneously measured the topography and surface potential (SP) of the film with Kelvin probe force microscopy (KPFM). The reduced areas appear as trenches in both the topographic and SP images (Fig. 4b and d), indicating decreased values after the reduction. Randomly distributed charge impurities were demonstrated to cause non-uniform distribution of the SP on reduced monolayer GO films deposited on dielectric substrates.^34^ Here the SP of the reduced 7-layer GO film is quite uniform, as thick films can effectively screen the impurity charges.

In KPFM measurements, the SP is known to be linked to WF based on the following relation:^35^

1 *ϕ*_s_ = *ϕ*_tip_ − eV_CPD_

*ϕ*_s_ and *ϕ*_tip_ is the WF of the sample and the KPFM tip, respectively. *V*_CPD_ refers to the measured SP. A reduced SP corresponds to an increased WF. Fig. 4 demonstrates, that higher the laser power, the lower the SP/the higher the WF.

To quantify the reduction effects, we performed a histogram analysis of the SP images, which are fitted with two Gaussian peaks (Fig. 4e–h), α and γ, corresponding to the SPs of the reduced GO films and bare Au films, respectively. The SPs of Au films vary slightly at different locations, possibly caused by surface contamination. The measured SP of the non-reduced GO film is −12.0 meV (ESI Fig. S6†). Upon reduction, the SP (WF) decreases (increases) by 0.5, 5.8, 10.1 and 17.3 mV, for the 0.8, 1.1, 1.4 and 1.7 mW laser irradiation, respectively. The WF of the reduced GO film increases linearly with laser power, with a gradient of 18.2 mV mW^−1^ (Fig. 4i). The 0.8 mW power caused very little change to the WF, though it reduced the film thickness by ∼2 nm. The WF of the few-layer GO films is known to be dependent on the film thickness, but saturates on thick films above 5 layers.^36^ This is possibly why the 0.8 mW laser almost made no change to the WF (as the reduced film is still above 5 layers), whereas higher powers reduce more substantially and hence impact the WF more significantly. Being able to fine tune the WF is crucial for achieving optimal performance for many electronic devices. For instance, in polymer solar cells,^37^ the WF of the hole/electron transporting layer must be close to the donor's HOMO/the acceptor's LUMO for the best performance; in field-effect transistors,^38^ lowering the Schottky barrier by reducing the WF difference between electrodes and semiconductors can significantly enhance device performance.

In conclusion, here we demonstrate that the reduction of individual GO films can be precisely controlled with laser irradiation. Such is the precision that the film thickness can be thinned with sub-nm resolution and the WF can be fine tuned with meV precision. The reduction is facilitated by extraordinary temperature gradients across the interlayers of GO films, which can be up to several hundred degrees per nanometer, which are easy to manipulate with laser powers. This provides important platforms for nanoscale thermodynamics investigations and paves the way towards precision nanotechnology, allowing to custom-design on-demand properties for optimal performance of devices.

## Experimental

### Sample preparation

A silicon wafer coated with 93 nm SiO_2_ was successively rinsed with acetone solution, IPA solution and deionized water, and blown dry with N_2_ gas. A 1 nm titanium adhesion layer and a 5 nm gold film were sputtered on top by physical vapor deposition (Kurt J. Lesker CMS-A DC magnetron sputterer), forming the specific contrast-enhancing substrate. GO flakes were prepared by dropcasting a diluted (concentration 4 × 10^−4^ mg mL^−1^) GO solution (purchased from Graphenea) on the substrate surface.

### Raman and optical measurement

A 532 nm laser of 0.8, 1.1, 1.4 and 1.7 mW was focused on the sample by using a 100× objective, NA = 0.9, Olympus MPLN100×BD. Raman signals were collected through a back-reflection configuration and measured with a Jobin Yvon HR640 Raman spectrometer. The intensities were normalized to the laser power. Optical contrast measurements were carried out with the 100× objective on a setup demonstrated in previous publications.^39^

### AFM and KPFM characterization

AFM measurements were taken with a Nanoscope IIIa (Model NS3a, Serial 1157, Digital Instruments) using a tapping mode and analysed with Nanoscope Analysis 1.5 software. KPFM measurements were taken with an Oxford Instruments MFP-3D Infinity device. A PtIr probe was used. 10 V ac at a frequency of 12 kHz (which is slightly lower than its resonance frequency) was applied between the probe and sample. The data was analysed with WSxM 4.0 software.^40^

### Theoretical simulation

The temperature distribution of GO layers under laser irradiation is computed through the finite element method provided by COMSOL Multiphysics. The 3D simulation model consists of GO thin films with different layers and Au, Ti, SiO_2_, and Si with a thickness of 5 nm, 1 nm, 93 nm and 100 nm, respectively. The cross section area of the simulation model is 5 ×5 μm^2^. The thermal properties of GO, SiO_2_ and Si used in the simulation are taken from previous studies. As the interface plays an important role in thermal transport, the interface thermal resistance is considered by adjusting thermal conductivity of materials through the serial model. As a result, the thermal conductivities of GO, Au, Ti, SiO_2_ and Si are set as anisotropic. A surface heat source with Gaussian distribution is adopted to simulate laser heating, which is applied at the center of the GO thin film. The unstructured tetrahedron meshes are constructed with the standard of "superfine".

## Conflicts of interest

There are no conflicts to declare.

## Supplementary Material

NA-002-D0NA00321B-s001

## References

[cit1] Chen D., Feng H., Li J. (2012). Chem. Rev..

[cit2] Pei S., Chen H. (2012). Carbon.

[cit3] Galande C., Gao W., Mathkar A., Dattelbaum A. M., Narayanan T. N., Mohite A. D., Ajayan P. M. (2014). Part. Part. Syst. Charact..

[cit4] Perrozzi F., Prezioso S., Ottaviano L. (2015). J. Phys.: Condens. Matter.

[cit5] Kim J., Cote L. J., Huang J. (2012). Acc. Chem. Res..

[cit6] Allen M. J., Tung V. C., Kaner R. B. (2010). Chem. Rev..

[cit7] Novoselov K. S., Fal'ko V. I., Colombo L., Gellert P. R., Schwab M. G., Kim K. (2012). Nature.

[cit8] Sokolov D. A., Morozov Y. V., McDonald M. P., Vietmeyer F., Hodak J. H., Kuno M. (2014). Nano Lett..

[cit9] Erickson K., Erni R., Lee Z., Alem N., Gannett W., Zettl A. (2010). Adv. Mater..

[cit10] Larciprete R., Fabris S., Sun T., Lacovig P., Baraldi A., Lizzit S. (2011). J. Am. Chem. Soc..

[cit11] Laracuente A. R., Dai Z., Marder S. R., Berger C., King W. P., de Heer W. A., Sheehan P. E., Riedo E. (2010). Science.

[cit12] Gómez-Navarro C., Meyer J. C., Sundaram R. S., Chuvilin A., Kurasch S., Burghard M., Kern K., Kaiser U. (2010). Nano Lett..

[cit13] Tu Y., Ichii T., Utsunomiya T., Sugimura H. (2015). Appl. Phys. Lett..

[cit14] Loh K. P., Bao Q., Eda G., Chhowalla M. (2010). Nat. Chem..

[cit15] Evlashin S., Dyakonov P., Khmelnitsky R., Dagesyan S., Klokov A., Sharkov A., Timashev P., Minaeva S., Maslakov K., Svyakhovskiy S., Suetin N. (2016). ACS Appl. Mater. Interfaces.

[cit16] Lu G., Park S., Yu K., Ruoff R. S., Ocola L. E., Rosenmann D., Chen J. (2011). ACS Nano.

[cit17] Lipatov A., Varezhnikov A., Wilson P., Sysoev V., Kolmakov A., Sinitskii A. (2013). Nanoscale.

[cit18] Wei Z., Wang D., Kim S., Kim S. Y., Hu Y., Yakes M. K., Laracuente A. R., Dai Z., Marder S. R., Berger C., King W. P., de Heer W. A., Sheehan P. E., Riedo E. (2010). Science.

[cit19] Eda G., Fanchini G., Chhowalla M. (2008). Nat. Nanotechnol..

[cit20] Zhang K., Fu Q., Pan N., Yu X., Liu J., Luo Y., Wang X., Yang J., Hou J. (2012). Nat. Commun..

[cit21] Stauber T., Peres N. M. R., Geim A. K. (2008). Science.

[cit22] Velický M., Hendren W. R., Donnelly G. E., Katzen J. M., Bowman R. M., Huang F. (2018). Nanotechnology.

[cit23] Perrozzi F., Prezioso S., Donarelli M., Bisti F., Marco P. D., Santucci S., Nardone M., Treossi E., Palermo V., Ottaviano L. (2013). J. Phys. Chem. C.

[cit24] Zhu Y., Murali S., Cai W., Li X., Suk J. W., Potts J. R., Ruoff R. S. (2010). Adv. Mater..

[cit25] Evlashin S. A., Svyakhovskiy S. E., Fedorov F. S., Mankelevich Y. A., Dyakonov P. V., Minaev N. V., Dagesyan S. A., Maslakov K. I., Khmelnitsky R. A., Suetin N. V., Akhatov I. S., Nasibulin A. G. (2018). Adv. Mater. Interfaces.

[cit26] Zhou Y., Bao Q., Varghese B., Ai L., Tang L., Tan C. K., Sow C., Loh K. P. (2009). Adv. Mater..

[cit27] Loh K. P., Bao Q., Eda G., Chhowalla M. (2010). Nat. Chem..

[cit28] Renteria J. D., Ramirez S., Malekpour H., Alonso B., Centeno A., Zurutuza A., Cocemasov A. I., Nika D. L., Balandin A. A. (2015). Adv. Funct. Mater..

[cit29] Sygellou L., Paterakis G., Galiotis C., Tasis D. (2016). J. Phys. Chem. C.

[cit30] Koh Y. K., Bae M. H., Cahill D. G., Pop E. (2010). Nano Lett..

[cit31] Tielrooij K. J., Hesp N. C. H., Principi A., Lundeberg M. B., Pogna E. A. A., Banszerus L., Mics Z., Massicotte M., Schmidt P., Davydovskaya D., Purdie D. G., Goykhman I., Soavi G., Lombardo A., Watanabe K., Taniguchi T., Bonn M., Turchinovich D., Stampfer C., Ferrari A. C., Cerullo G., Polini M., Koppens F. H. L. (2018). Nat. Nanotechnol..

[cit32] Emelianov A. V., Kireev D., Offenhäusser A., Otero N., Romero P. M., Bobrinetskiy I. I. (2018). ACS Photonics.

[cit33] Wei P., Bao W., Pu Y., Lau C. N., Shi J. (2009). Phys. Rev. Lett..

[cit34] Giusca C. E., Perrozzi F., Ottaviano L., Treossi E., Palermo V., Kazakova O. (2015). Carbon.

[cit35] Negishi R., Takashima K., Kobayashi Y. (2018). Jpn. J. Appl. Phys..

[cit36] Jaafar M., Lopez-Polin G., Gomez-Navarro C., Gomez-Herrero J. (2012). Appl. Phys. Lett..

[cit37] Stratakis E., Savva K., Konios D., Petridisa C., Kymakis E. (2014). Nanoscale.

[cit38] Liu W., Kang J., Sarkar D., Khatami Y., Jena D., Banerjee K. (2013). Nano Lett..

[cit39] Katzen J. M., Velický M., Huang Y., Drakeley S., Hendren W., Bowman R. M., Cai Q., Chen Y., Li L., Huang F. (2018). ACS Appl. Mater. Interfaces.

[cit40] Horcas I., Fernandez R. (2007). Rev. Sci. Instrum..

