# Supplementary material for: Customizing the reduction of individual graphene oxide flakes for precise work function tuning with meV precision
Source: Nanoscale Adv. 2020 Jun 1;2(7):2738–44. doi: 10.1039/d0na00321b (PMC9419187; doi:10.1039/d0na00321b)
Supplement: NA-002-D0NA00321B-s001 [file NA-002-D0NA00321B-s001.pdf]

Electronic Supplementary Material (ESI) for Nanoscale Advances.
This journal is © The Royal Society of Chemistry 2020

Supplementary Information

Customizing the Reduction of Individual Graphene Oxide Flakes for Precise Work Function Tuning with meV Precision

*Yuefeng Huang,[1] Dengke Ma,[2] Patrick Turner,[1] Gavin E Donnelly,[1] Joel M Katzen,[1] William R Hendren,[1] Marty J Gregg,[1] Robert M Bowman,[1] Lifa Zhang[2], Gang Zhang,[3] Fumin Huang[1]**

[1]School of Mathematics and Physics, Queens University Belfast, Belfast, BT7 1NN, UK
[2]NNU-SULI Thermal Energy Research Center (NSTER) and Center for Quantum Transport and Thermal Energy Science (CQTES), School of Physics and Technology, Nanjing Normal University, Nanjing, 210023, China
[3]Institute of High Performance Computing, A*STAR, Singapore 138632, Singapore

*E-mail: f.huang@qub.ac.uk

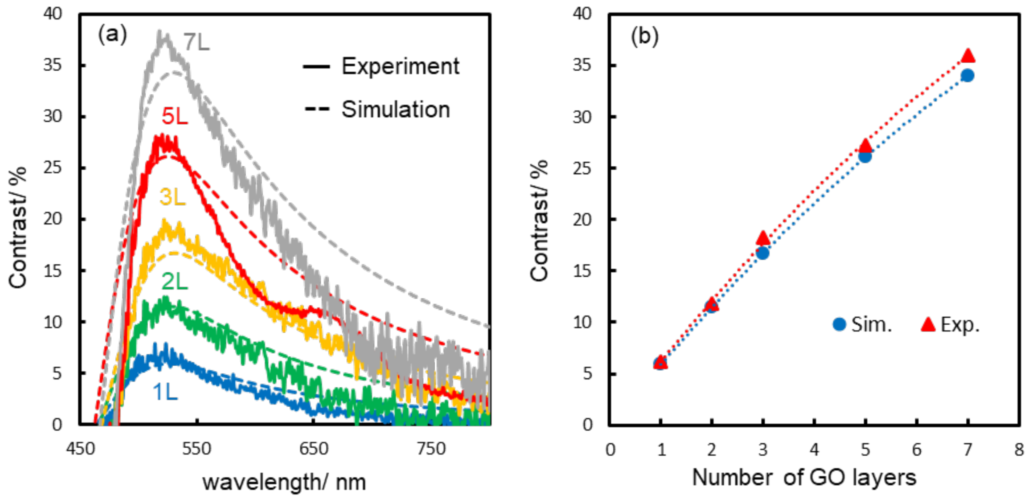

Figure S1 Optical contrast spectroscopy of few-layer graphene oxide films. (a) Measured (solid curves) and simulated (dashed curves) contrast spectroscopy of graphene oxide films of various thickness, which match each other quite well. The anomalous shape of 5L is possibly due to defects on the film. (b) Maximum contrast vs number of layers, for simulated (circles) and experimental (triangles) results. Graphene oxide films are deposited on a stratified multilayer structure, composed of 5nm Au, 1 nm Ti, 93 nm SiO_2 and Si substrate.

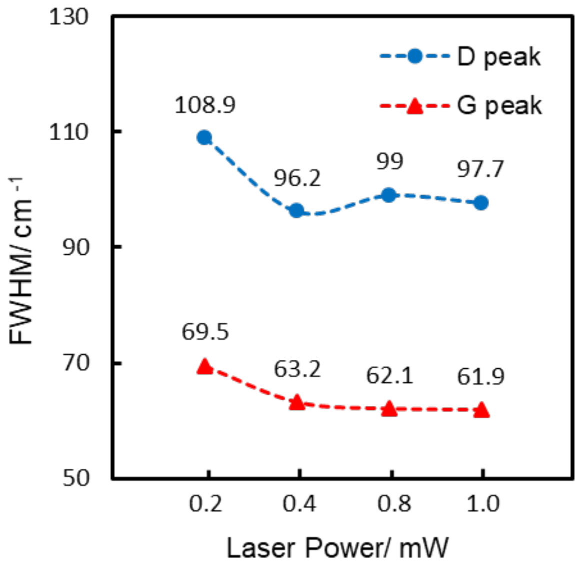

Figure S2 The full-width-at-half-maximum (FWHM) of the D-mode and G-mode of the Raman signals of a bilayer graphene oxide film, successively irradiated by various laser powers of 0.2, 0.4, 0.8 and 1.0 mW. The excitation laser is 532 nm, focused by a 100× objective, NA=0.9, Olympus MPLN100×BD, for a duration of 60 s of each laser power. Raman spectra were measured immediately after the laser irradiation.

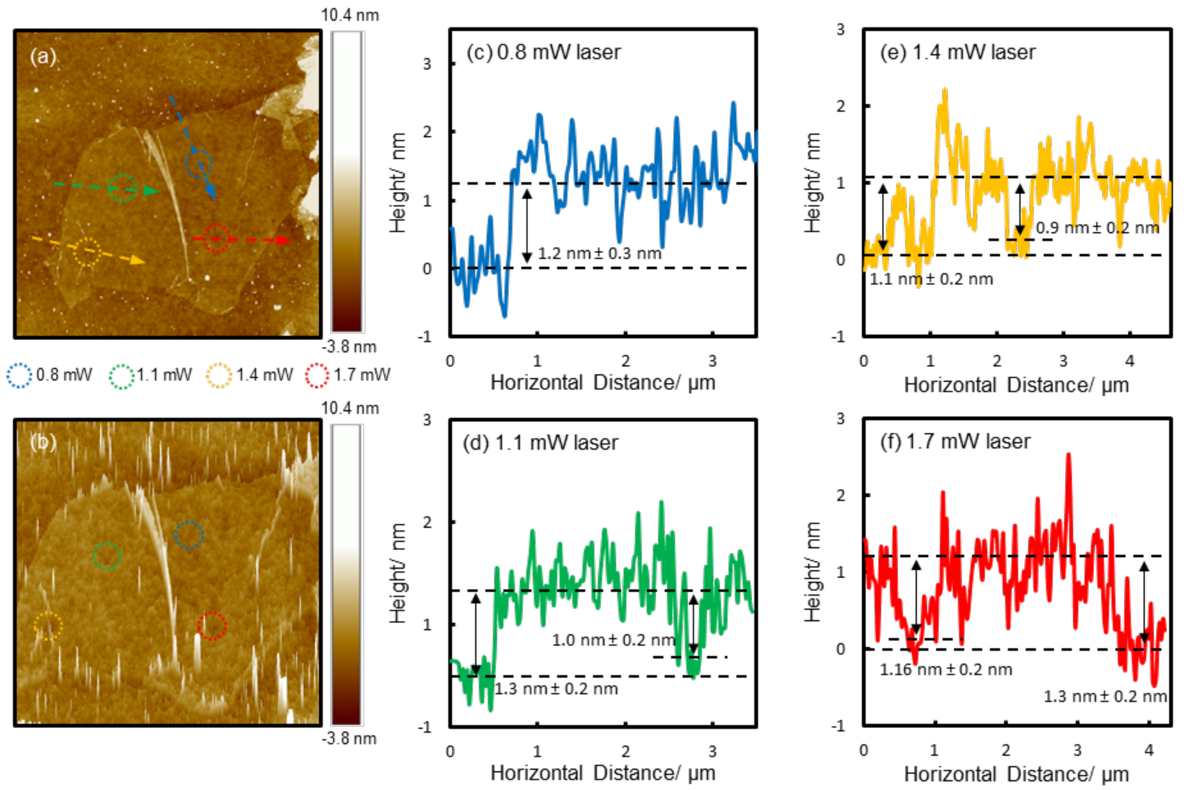

Figure S3 (a) AFM topography image of a monolayer graphene oxide film, irradiate by various laser powers (irradiated laser spots are marked by circles of different colors for different laser powers). Scale bar: 3 μm. (b) 3D plot of (a). (c-f) The height profiles along the dashed lines indicated in (a). Dips indicate the reduced film thickness.

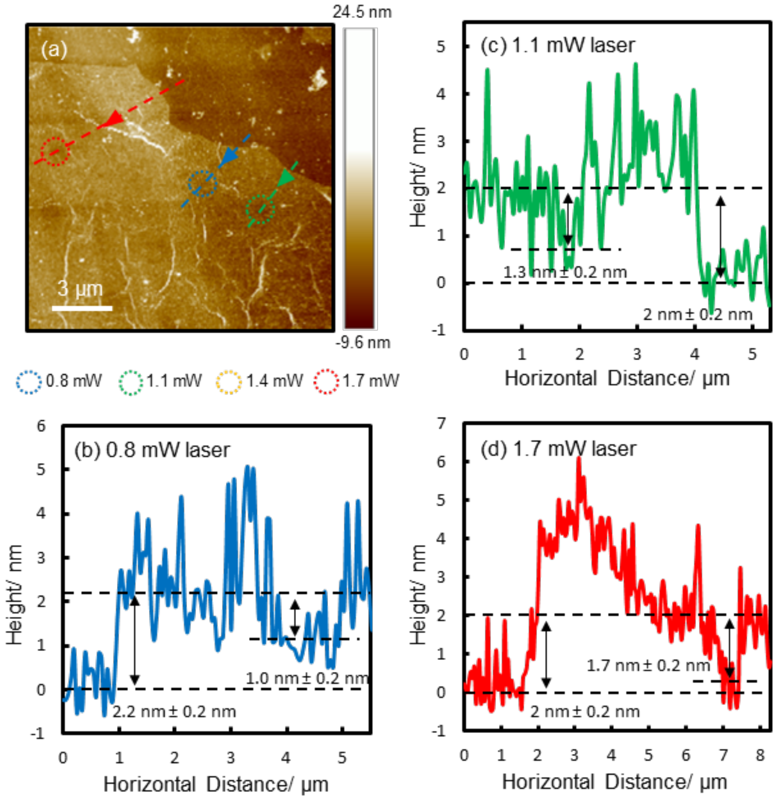

Figure S4 (a) AFM topography image of a bilayer graphene oxide film, irradiate by various laser powers (laser spots are marked by circles of different colors for different laser powers). (b-d) The height profiles along the dashed lines indicated in (a). Dips indicate the reduced film thickness.

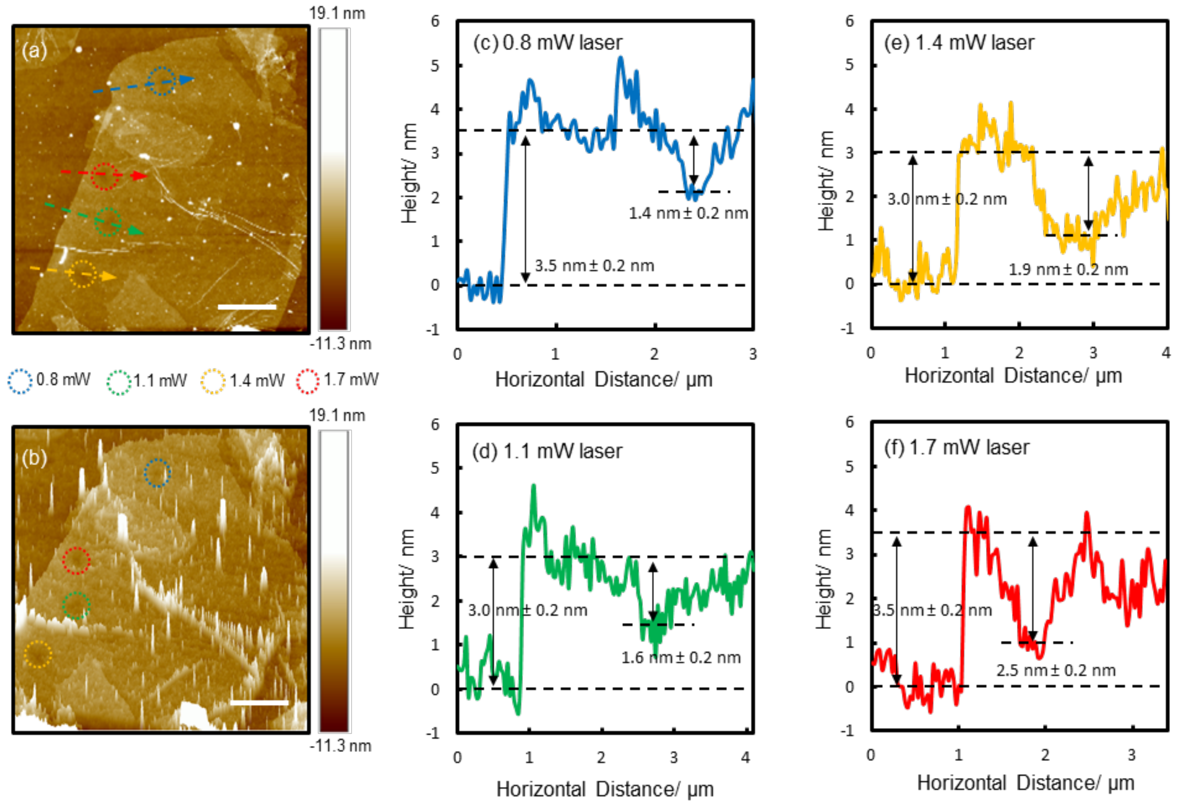

Figure S5 (a) AFM topography image of a three-layer graphene oxide film, irradiated by various laser powers (laser spots are marked by circles of different colors for different laser powers). Scale bar: 3 μm. (b) 3D plot of (a). (c-f) The height profiles along the dashed lines indicated in (a). Dips indicate the reduced film thickness.

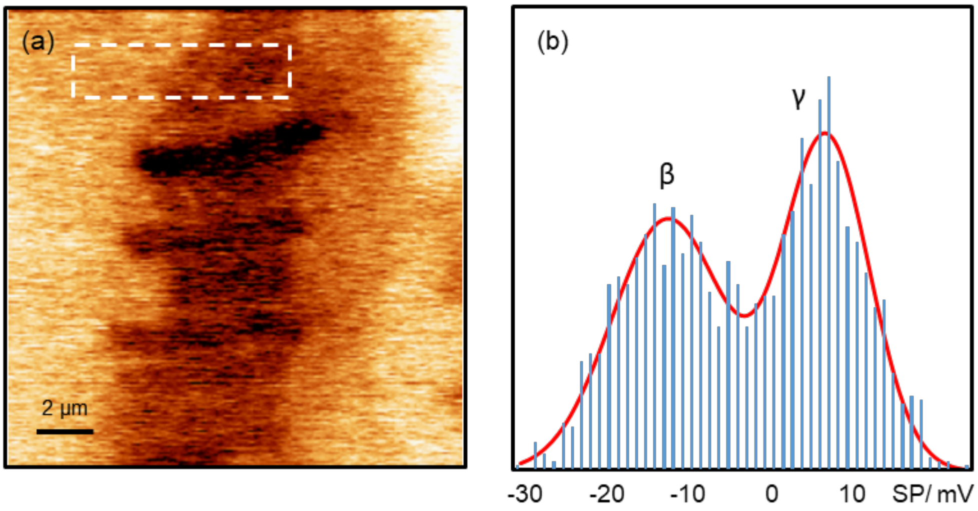

Figure S6 (a) The surface potential (SP) image of a 7-layer GO film. (b) Histogram analysis of the measured SP image on an area without being irradiated by laser (highlight by the dashed box), which are fitted with two Gaussian peaks corresponding to the surface potential of GO film (β) and Au film (γ), respectively. The surface potential of the non-reduced GO film is -12 mV (β peak).
